# Comment on “Yield of Screening Colonoscopy in Renal Transplant Candidates”

**DOI:** 10.1155/2016/5824235

**Published:** 2016-03-31

**Authors:** Amelie Therrien, Mickael Bouin

**Affiliations:** Centre de Recherche du Centre Hospitalier de l'Université de Montréal, Service de Gastroentérologie, Hôpital Saint-Luc, Université de Montréal, 1058 Saint-Denis Street, Montréal, QC, Canada H2X 3J4

To the editors of the Canadian Journal of Gastroenterology and Hepatology, We read with great interest the article “Yield of Screening Colonoscopy in Renal Transplant Candidates” by AlAmmel and colleagues. This study about the results of screening colonoscopy in a cohort of 169 renal transplant candidates over 50 years old showed a prevalence of colorectal polyps of 24%, 4 advanced adenomas, and 1 adenocarcinoma. The authors conclude that colorectal cancer screening is indicated in renal transplant candidates over 50 years old and that the choice of the screening test should be individualized based on patient's preference and risk benefit ratio [1].

We previously published a retrospective study about the results of pretransplant colonoscopies in renal transplant recipients from January 2007 to December 2009 at the Centre Hospitalier de l'Université de Montréal [2]. Any pretransplant colonoscopy in the five years preceding the transplant was included. On 64 pretransplant colonoscopies, 45 (70.3%) were for screening purposes. 15 (33.3%) of these individuals had polyps and the prevalence of adenomas and advanced adenomas was 24.4 and 6.7%, respectively. More importantly, 8 (53.3%) of these subjects had at least one lesion proximal to the splenic angle. Our cohort with screening colonoscopies included 11 patients aged less than 50 years and 3 of them (27.3%) had adenomas, an even higher rate than the overall cohort. No major adverse events occurred; two episodes of bleeding associated with polyp removal were immediately treated endoscopically.

Our results are in accordance with the results from AlAmmel and colleagues, reinforcing the need of colorectal cancer screening among renal transplant candidates, even if aged less than 50 years. It has indeed been proven that the incidence of colorectal cancer is higher among solid organ recipients as soon as two years after transplant, notably in individuals less than 50 years old [3, 4]. The low sensitivity of the faecal immunochemical testing for advanced neoplasia in this population as mentioned by AlAmmel and colleagues, the high prevalence of proximal lesions demonstrated in our study, and the high standardized incidence ratio of right colon neoplasia in post-solid organ transplant population established in a Swedish cohort study [5] provide compelling arguments for total colonoscopy as the screening test of choice.
